# Dimensions of Peer Sexual Harassment Victimization and Depressive Symptoms in Adolescence: A Longitudinal Cross-Lagged Study in a Swedish Sample

**DOI:** 10.1007/s10964-016-0446-x

**Published:** 2016-02-24

**Authors:** Heléne Zetterström Dahlqvist, Evelina Landstedt, Robert Young, Katja Gillander Gådin

**Affiliations:** Department of Health Sciences, Mid Sweden University, 851 70 Sundsvall, Sweden; Department of Public Health and Clinical Medicine, Umeå University, 901 87 Umeå, Sweden; MRC/CSO Social and Public Health Sciences Unit, University of Glasgow, 200 Renfield Street, Glasgow, G2 3QB UK

**Keywords:** Sexual harassment, Depressive symptoms, Adolescence, Directional pathways, Gender differences

## Abstract

Sexual harassment is commonly considered unwanted sexual attention and a form of gender-based violence that can take physical, verbal and visual forms and it is assumed to cause later depression in adolescents. There is a dearth of research explicitly testing this assumption and the directional pathway remains unclear. The purpose of this study was to use a feminist theoretical framework to test competing models in respect of the direction of the relationships between dimensions of peer sexual harassment victimization and dimensions of depressive symptoms from ages 14 to 16 in adolescents. The study also aimed to investigate gender differences in these pathways. Cross-lagged models were conducted using a three-wave (2010, 2011 and 2012) longitudinal study of 2330 students (51 % females) from Sweden, adjusted for social background. Girls subjected to sexual harassment in grade seven continued to experience sexual harassment the following 2 years. There was weaker evidence of repeated experience of sexual harassment among boys. Depressive symptoms were stable over time in both genders. Sexual name-calling was the dimension that had the strongest associations to all dimensions of depressive symptoms irrespective of gender. In girls, name-calling was associated with later somatic symptoms and negative affect, while anhedonia (reduced ability to experience pleasure) preceded later name-calling. Physical sexual harassment had a reciprocal relationship to somatic symptoms in girls. In boys, name-calling was preceded by all dimensions of depressive symptoms. It is an urgent matter to prevent sexual harassment victimization, as it is most likely to both cause depressive symptoms or a reciprocal cycle of victimization and depression symptoms in girls as well as boys.

## Introduction

One of the most urgent public health issues in the majority of Western countries, including Sweden, is poor mental health such as depressive symptoms and psychological distress in adolescence (World Health Organization 2015). This has increased substantially over the last three decades (Hagquist 2010; Bremberg 2015; Bor et al. 2014; Collishaw et al. 2010; Sweeting et al. 2009) and the World Health Organization (WHO) states that depression is the worldwide leading cause of illness and disability in adolescents (World Health Organization 2015). Factors associated with poor mental health in young people include: socioeconomic influences (Reiss 2013), including personal relative affluence (Zetterström Dahlqvist et al. 2012) and parental unemployment (Myklestad et al. 2011); familial factors such as maternal depression (Goodman et al. 2011), disrupted family (Carlsund et al. 2013), and parental immigrant background (Zetterström Dahlqvist et al. 2012; Carlerby et al. 2011), biopsychosocial factors such as pubertal timing (Angold and Costello 2006; Skoog et al. 2015); poor social relationships (Olsson 2011), and low self-esteem (Sowislo and Orth 2013). Interpersonal violence and harassment, such as physical and dating violence, bullying and sexual harassment have also been strongly implicated as risk factors for poor mental health (Gruber and Fineran 2008; Espelage and Holt 2007; Bucchianeri et al. 2014; Zetterström Dahlqvist et al. 2012; Landstedt and Gillander Gådin 2011). The body of research on the mental health outcomes of bullying in school is considerable (Hansen et al. 2012), while research on the impact of sexual harassment is scarcer. The existing studies of the relationship between sexual harassment and depressive symptoms are typically cross-sectional (Bucchianeri et al. 2014; Gruber and Fineran 2008) or lack a longitudinal study design that can be used to infer directions of associations e.g. (Skoog et al. 2015; Chiodo et al. 2009; Goldstein et al. 2007; Felix and McMahon 2007a). Recognizing this gap in our understanding of the directional pathways between sexual harassment and depressive symptoms in adolescents, the current longitudinal study investigates the role of long term sexual harassment victimization in the development of adolescent depressive symptoms.

### Sexual Harassment Victimization and Poor Mental Health

Longitudinal studies show that sexual harassment victimization strongly predicts future emotional distress, depressive symptoms, problem substance abuse, and adjustment difficulties, but that gender is an inconsistent moderator of this relationship. Some studies report the association for both genders (Chiodo et al. 2009; Felix and McMahon 2007a), while others find the link among females only (Goldstein et al. 2007). In a 1 year follow-up multi-group path model, Skoog et al. (2015) found that sexual harassment victimization mediated the link between early pubertal timing and depressive symptoms in 13-year-old girls, but not in those who were 1 year older. However, the authors acknowledge that this design cannot address the direction of effects. To our knowledge, only one longitudinal study explicitly modeled the directional direction of sexual harassment victimization and adverse mental health outcomes (Marshall et al. 2013). Marshall et al. (2013) used separate Swedish and Canadian datasets to determine the key directional pathways between sexual harassment and deliberate self-injury and the moderating role of gender. Results of the Swedish data suggested a bidirectional relationship, which was strongest among girls, and which showed that adolescents who were harassed were likely to subsequently self-injure, and, in turn, those who self-injured were subsequently likely to be harassed. Mediation analysis suggested that these were at least two distinct groups of adolescents, presumably with divergent psychosocial and socio-demographic risks. Analysis of the Canadian sample suggested that deliberate self-injury predicted subsequent sexual harassment irrespective of gender, but not vice versa. One interpretation of these seemingly conflicting findings is that adolescent Swedish girls, but not boys, with mental health problems may be particularly vulnerable to sexual harassment, while in Canada, irrespective of gender, sexual harassment perpetrators may see peers displaying signs of poor mental health as easy targets. However, the methodologies differ between the Swedish and the Canadian cohorts in this study (Marshall et al. 2013), which could account for these cross-country differences. In the light of the overall results by Marshall et al. (2013), it is important to further investigate the directional relationship between sexual harassment victimization and poor mental health outcomes. Victimization may not necessarily precede the mental health outcome—emotional vulnerability such as depressive symptoms may very well make students vulnerable for victimization as they may be perceived as “easy targets” by harassers.

### Perspectives on Sexual Harassment

Sexual harassment is considered a form of gender-based violence (Heise et al. 1999; Stein 1995, 1999), and is commonly defined as unwanted or unwelcome sexual attention. Sexual harassment victimization in adolescence is common, and the lifetime prevalence varies considerably depending on age and setting, but it has been reported to be between 45–88 % in male and 52–96 % in female students (Ormerod et al. 2008; Felix and McMahon 2007b; AAUW 2001; Gruber and Fineran 2008; Zetterström Dahlqvist et al. 2012). A longitudinal study (Chiodo et al. 2009) showed that sexual harassment victimization in adolescence is relatively stable over time (in both genders), and that victimization at age 14 is strongly associated with other forms of relationship violence two and a half years later. According to two previous studies, victimization increases throughout the early life course; age 10–14 (Petersen and Hyde 2009), and age 11–13 (McMaster et al. 2002). Sexual harassment in school is in fact so common that students consider it a part of everyday life at school and something you “just have to learn to deal with” (deLara 2008; Robinson 2005; Hlavka 2014).

A common discourse of sexual harassment is that perpetration is due to individual behavior problems or a lack of social skills, such as immature interactions with the opposite sex, (see e.g. McMaster et al. 2002; Petersen and Hyde 2009). A poststructuralist feminist theory framework seeking to understand the phenomenon of adolescent (as well as adult) sexual harassment as a sociocultural construction of gender, sexuality, and power relations (Conroy 2013; Kimmel 2008; Robinson 2005; Keddie 2009), could offer a different discourse of sexual harassment. Robinson (2005), for example, argues that sexual harassment perpetration is rarely about an individual’s problems or lack of social skills as suggested by e.g. McMaster et al. (2002). Instead, Robinson (2005) stresses that sexual harassment is constituted within a broader social context of values and power relationships operating around gender and sexuality also present in the broader society. The predominant values in most societies are usually the dominance of the prevailing hegemonic (heterosexual) masculinity, which is reflected and reproduced in school (Jones 1985; Connell 1987; Hlavka 2014). Robinson (2005) and Chambers et al. (2004), argue that sexual harassment in school is a tool to assert male dominance over females, and also a tool for policing heterosexual masculinity within male peer groups. Furthermore, scholars have argued that sexual harassment is not necessarily about sexual interest on behalf of the perpetrator (Mahony 1989; Jones and Mahony 1989), but rather about power issues. Robinson (2005) has shown, for example, how boys use a range of sexual harassment practices to put girls “back in their place” when power relations are challenged. In addition, Felix and McMahon (2007a) have shown that being victimized by a girl had no impact whatsoever on internalizing or externalizing behaviors in boys or in girls. We argue that understanding how sexual harassment is used as a tool to establish asymmetric power between boys and girls, as well as between boys, may assist in interpreting directional associations between sexual harassment victimization and depressive symptoms. Furthermore, sexual harassment may take physical, verbal or visual forms (AAUW 2001), but, depending on items used to measure sexual harassment, other dimensions (e.g., hostile environment) have been proposed in adolescent populations (Vega-Gea et al. 2016; McMaster et al. 2002; Witkowska and Kjellberg 2005). In order to inform future preventive measures, investigating the directional pathways of dimensions of sexual harassment and depressive symptoms in this age group makes it possible to obtain information about what behaviors in more detail that are associated with what dimensions of depressive symptoms.

## The Current Study

As the current study focuses on victimization and not on perpetration we suggest, in accordance with Conroy (2013), Robinson (2005), and Chambers et al. (2004) that a feminist approach that consider sexual harassment as an assertion of male dominance and asymmetric power relations would contribute to the understanding of (a) who is being victimized by what dimension of sexual harassment, (b) how dimensions of depressive symptoms are associated with different dimensions of sexual harassment over time, and (c) how gender influences such associations. We therefore identified a need to clarify the direction of the associational pathways between dimensions of peer sexual harassment victimization and dimensions of depressive symptoms during adolescence, as well as how gender influences these pathways. Accordingly, the aim of the current study was to test competing models in respect of the direction of the relationships between dimensions of peer sexual harassment victimization and dimensions of depressive symptoms from ages 14 to 16 in adolescents using accelerated cross-lagged panel analysis and to investigate gender differences in these pathways. Specifically, we specified four theoretical hypotheses for the analytical models. Sexual harassment victimization was hypothesized to be stable over time since the assertion of male dominance and asymmetric power relations is not expected to change over time in this age group as these gender relations are a reflection of the broader society (Connell 1987). Furthermore, these gender relations continue into adulthood (McDonald 2012). Also, based on previous research, the stability of depressive symptoms over time was expected to be higher in girls than boys (Cole et al. 2009). High levels of our dimension of sexual harassment victimization was hypothesized to precede increases in the levels of each dimensions of depressive symptoms if sexual harassment were associated with establishing asymmetric power relations between genders. Furthermore, indicators of vulnerability in potential victims such as high levels in each dimension of depressive symptoms were hypothesized to precede increases in levels of each dimension of sexual harassment victimization. This is the pattern we would expect if sexual harassment is associated with reinforcing hegemonic (heterosexual) masculinity and reinforcing asymmetric power relations between the genders, but also within male peer networks where vulnerability is seen as less masculine. Lastly, the relationship between dimensions of sexual harassment victimization and dimensions of depressive symptoms were hypothesized to be bidirectional if both establishing and reinforcing asymmetric power relations are a phenomena in this age group.

## Methods

### Context

We utilized data from the Youth Health Development-project (YHD-project), a longitudinal study of health development in adolescents in the Northern part of Sweden, which included a questionnaire about the psychosocial school environment. The municipality in which the YHD-project was implemented is of medium size (60,000 inhabitants). It is characterized by a diverse socioeconomic base with a focus on tourism and small- and medium-sized enterprises. Compared to Sweden in general, the municipality has fewer inhabitants with foreign backgrounds (Statistics Sweden 2010). In Sweden, most children start school in the fall term when they reach the age of seven and attendance is compulsory for all children up to the age of 16. Compulsory school is free of charge. In the current study, grade levels seven to nine (ages 14–16) are referred to as high school.

### Participants and Procedure

Data were collected using an electronic questionnaire which was administered in three Waves; Wave 1 in January 2010, Wave 2 in January 2011, and Wave 3 in January 2012. All public (N = 9) and one out of four independent high schools with students in grades seven to nine participated in the study. The electronic questionnaire was built with the Easy Research software and was filled in on computers during school hours and a research assistant was present in the classroom. Informed consent was obtained from parents as well as students. Students were informed about the aims of the questionnaire and that they could withdraw from participation at any time.

The response rate of the total sample, including both genders in grade seven to nine, was 81.9 % at Wave 1 (2010), 80.49 % at Wave 2 (2011) and 79.51 % at Wave 3 (2012). In Wave 1, 402 students were in grade seven. The attrition to follow up 1 year later (Wave 2) was 16.7 and 27.4 % 2 years later (Wave 3). The following differences exist between those lost to attrition and those who were not lost: Wave 1–Wave 2: More boys lost to attrition (n = 38) reported living in a disrupted family, *p* = .004. Wave 2–Wave 3: There was lower mean scoring on sexual harassment victimization in boys lost to attrition (n = 20), *p* = .011. In girls lost to attrition (n = 50), there was higher mean scoring on sexual harassment victimization, *p* = .004. The total sample consisted of 2342 students of whom 1146 were male and 1196 were female. Accelerated cross-lagged panel analysis uses cases with missing data, however because this type of analysis models changes between grades, twelve students who either advanced or were held back a grade were excluded reducing the final sample to 1192 girls and 1138 boys.

### Measures

#### Depressive Symptoms

Depressive symptoms were measured using the Center for Epidemiological Studies Depression Scale (CES-D) (Radloff Sawyer 1977) developed for screening purposes (Radloff Sawyer 1977, 1991; Eaton et al. 2004). This scale has a range of 0–60 with higher scores indicating higher levels of depressive symptoms. We used a three factor solution as suggested by Carleton et al. (2013) and our data fit with the same three dimensions as in their study; somatic symptoms, negative affect and anhedonia. All dimensions had acceptable internal consistency (Cronbach’s alphas): Somatic symptoms: Grade 7, 0.79; Grade 8, 0.81; and Grade 9, 0.80. Cronbach’s alphas for negative affect were: Grade 7, 0.84; Grade 8, 0.84; and Grade 9, 0.85. Cronbach’s alphas for anhedonia were: Grade 7, 0.71; Grade 8, 0.74; and Grade 9, 0.77.

#### Sexual Harassment

Sexual harassment was measured using fourteen items previously used in the Gruber and Fineran (2007, 2008) studies as well as in the AAUW study (2001). Respondents were asked to indicate how often they had experienced the following behaviors against their will during the past 6 months (never, once, a few times, many times): touched, grabbed or pinched you in a sexual manner; pushed you into a corner in a sexual manner; spread sexual rumors about you; commented on you, made a joke out of you or gesticulated in your direction in a sexual manner; looked at you in a manner that felt intrusive and sexual; shown or left you sexual images, photos or drawings; written sexual messages about you on bathroom walls or in locker rooms; called you a lesbian, fag or such words; pulled/pulled off your clothes in a sexual manner (e.g. pulled your bra straps, pulled your underwear or pulled up your skirt); tried to kiss or hug you; called you a “four letter word”; commented on your looks, your body or your personal life in a sexual manner; spread comments about you or pictures of you on mobile phones; publicly commented on how attractive or unattractive you are. This scale has a range of 14–56 with higher scores indicating higher levels of sexual harassment victimization and demonstrates good reliability in the current sample (α = .79–.88). McMaster et al. (2002) suggest a three factor solution (verbal, physical, and visual) while Vega-Gea et al. (2016) suggest a two dimensional solution (verbal–visual and physical). Witkowska and Kjellberg (2005) propose a nested structure with a general sexual harassment factor and two specific factors (hostile environment and sexual attention) for female but not for male students for whom only a general sexual harassment factor had an acceptable fit. As these authors have not used the exact same items that are being used in the current study, we conducted an Exploratory Factor Analysis within Mplus using quatermin and varimax rotation. Results suggested a three factor solution which was stable across grades using 8 of the 14 original items (details available upon request). Three dimensions: Physical (4-items), verbal name-calling (2-items), and public display (2-items). Internal consistency (Cronbach’s alphas) of these three dimensions were satisfactory; physical harassment: Grade 7, 0.83; Grade 8, 0.84, and Grade 9, 0.82. Cronbach’s alphas of verbal name-calling were: Grade 7, 0.83; Grade 8, 0.81, and Grade 9, 0.80. Public display: Grade 7, 0.87; Grade 8, 0.82; Grade 9, 0.77.

### Covariates

#### Parental Employment Status

Parental unemployment has been shown to be a risk factor for psychological distress (Myklestad et al. 2011) and is therefore used as a covariate in this analyses. At baseline students were asked “Does your [mother/father] have a job?” Response options were “Yes”; “No”; “I don’t know”; “I do not have a [mother/father] or do not see [her/him]”. Those from families where both parents were employed were contrasted with those in which one or both parents were unemployed.

#### Family Composition

A disrupted family situation has been shown to be a risk factor for multiple health complaints and low well-being in Swedish adolescents (Carlsund et al. 2013) and may also influence the development of depressive symptoms. At baseline students were asked “With whom do you live? (You may mark multiple choices)”. Response options were “Both my mother and father”; “Sometimes my mother, sometimes my father”; “Mother with new partner”; “Father with new partner”; “Only my mother”; “Only my father”; “Other/s”. Those who lived with both parents at baseline were contrasted with those who did not.

#### Parental Immigrant Background

No questions on race/ethnicity of the students were included in the YHD-study. However, in a Swedish context, parental migrant background has been shown to be associated with depressive symptoms (Zetterström Dahlqvist et al. 2012) and subjective health complaints (Carlerby et al. 2011). Hence, migrant family background was used as a covariate. At baseline students were asked “In what country was your [mother/father] born?” Response options were “Sweden”; “In another country (which?)”; and “I don’t know”. Those with one or both parents from a non-Swedish background were classified as having a migrant family background.

#### Personal Relative Affluence

In Wave 2 of the larger YHD-study, personal relative affluence has been shown to be strongly (inversely) associated to depressive symptoms in both genders (Zetterström Dahlqvist et al. 2012). At baseline, personal relative affluence was assessed by a question on how often (always, often, sometimes, rarely, never) in the past 6 months the respondents have had enough money to be able to do the same things as their friends. The variable has been treated as continuous.

### Statistical Analyses

The primary method of analysis was a robust full maximum-likelihood estimation (RFMLE) within a cross-lagged structural equation modeling framework using accelerated cohort analysis. Traditional cross-lagged analysis allows only data from students who participated in all three waves to contribute to longitudinal analysis, i.e. only those first surveyed in 2010 in grade seven and followed up in 2011 and 2012. In the current analyses data was converted from wave (year of collection) to grade (age at data collection) using the DATA COHORT command in the MPLUS statistical software package (Muthén and Muthén 1998–2012). This maximizes the amount of data available and focuses on age differences in longitudinal pathways. The accelerated panel method capitalizes on the overlapping waves of data to provide unbiased estimates of directional pathways using all available data (Little et al. 2007). Preliminary analyses suggested no substantive cohort effects, which otherwise can bias estimates from accelerated panel designs.

**Fig. 1:**
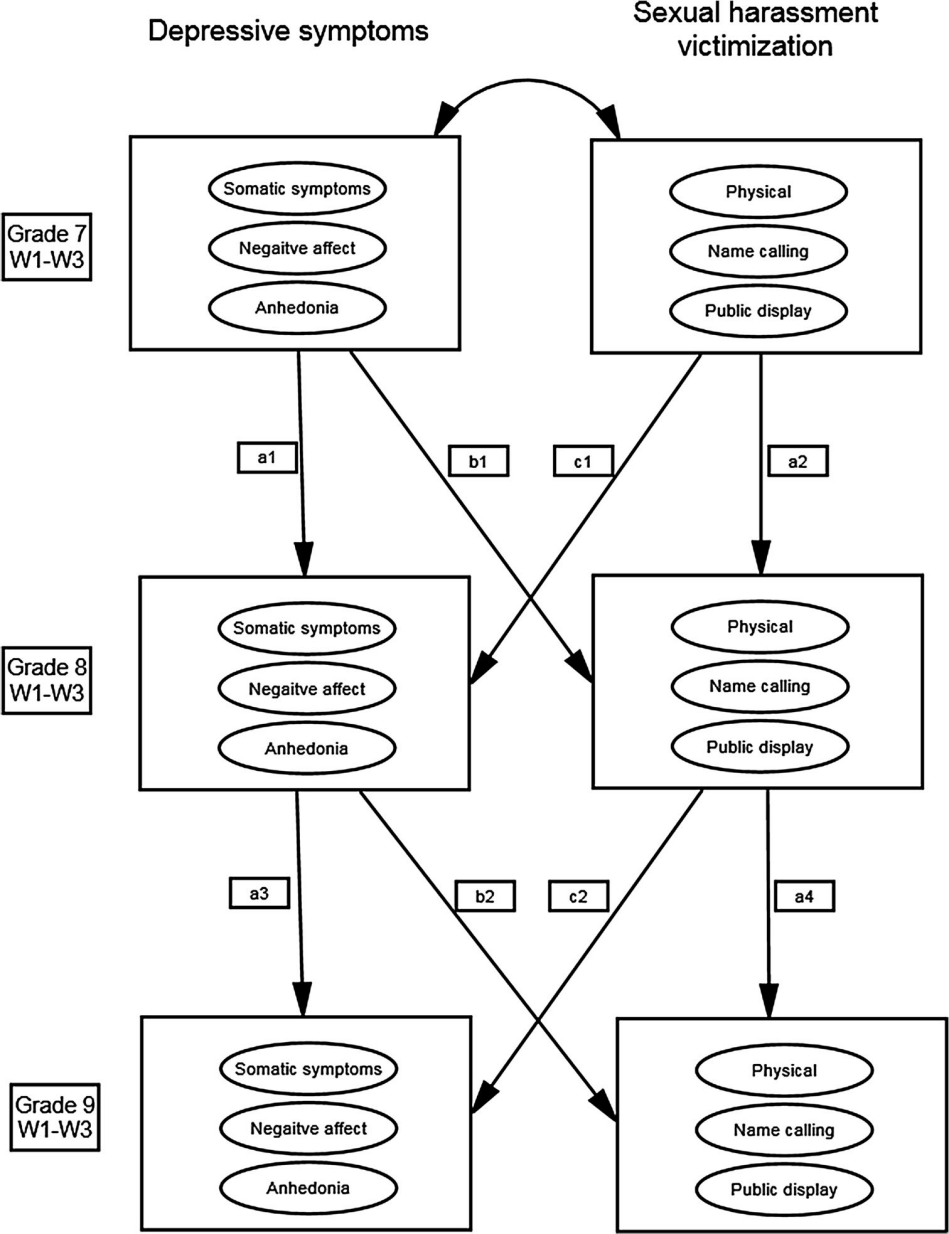
Hypothesized models of the relationship between dimensions of sexual harassment victimization and dimensions of depressive symptoms between Grade 7 and 9. (Symbols of error terms of the manifest variables have been omitted to avoid clutter). *Models* “stability” over time = a paths a only; lagged sexual harassment victimization to depressive symptoms (SH → DS) = a + b; lagged depressive symptoms to sexual harassment victimization (DS → SH) = a + c; lagged bidirectional = a + b + c

Gender differences in levels of dimensions sexual harassment, dimensions of depressive symptoms and covariates were determined using *t* tests (using RFMLE to adjust for missing data) and Chi square tests as appropriate. The strength of associations between variables was assessed by Pearson correlations (also using RFMLE). Cross-lagged path analysis model (under certain assumptions) the directional relationships between two or more variables across time. The fit of four cross-lagged models of the relationship between dimensions of sexual harassment and depressive symptoms from grades seven, eight and nine were compared. The first baseline model, hence forth called the ‘stability model’, hypothesized that earlier depressive symptoms predict later depressive symptoms and earlier sexual harassment later harassment, but that there is no relationship between depressive symptoms and sexual harassment (see Fig. 1). The second lagged sexual harassment to depressive symptoms model, henceforth the ‘SH → DS model’ expands on the stability model by hypothesizing a directional relationship from earlier sexual harassment to later depressive symptoms, but not vice versa. The third model lagged depressive symptoms to sexual harassment hypothesized relationships from earlier depressive symptoms to later sexual harassment, but not vice versa (henceforth the “DS → SH model”). The fourth cross-lagged bidirectional model, which we labelled the “bidirectional model”, combined the two, hypothesizing relationships from earlier depressive symptoms to later victimization and vice versa (a reciprocal relationship).

Cross-lagged path analysis with latent variables was conducted using Mplus 7.3, with separate cross-lagged models for each gender and adjusted for covariates. Contemporaneous disturbances between depressive symptoms and sexual harassment latent variables were correlated to account for the effect of un-modelled causal variables. Covariates were allowed to co-vary with each other and errors from identical manifest scale items were allowed to correlate across grades. To address missing data and non-normality in the data models were estimated using RFMLE. Standard measures of fit were used to compare the stability model with all other models. Fit measures included: a global fit measure, the comparative fit index (CFI), range 0–1 with a CFI of above 0.90 considered acceptable (Bentler 1990) and a CFI above 0.95 considered optimal (Hu and Bentler 1999); a residual fit statistic, the root mean square error of approximation (RMSEA), range 0–1 with a RMSEA below 0.05 considered good (Browne and Cudeck 1993). The Satorra–Bentler scaled Chi square difference test was used to formally test statistical differences between the models. Standardized path coefficients were used to informally assess the magnitude and relative importance of the pathways. Multi-groups analysis was used to formally compare male and female models by constraining stability and cross-lagged pathways to be equal between genders.

## Results

### Descriptive Statistics and Correlations

Descriptive statistics for the covariates by gender are provided in the bottom of Table 1 and shows that there were no gender differences in covariates at baseline. Females reported significantly higher depressive symptoms than males in six out of nine comparison across all the three dimensions/subscales. There were no significant gender differences regarding anhedonia symptoms. Females reported significant higher levels of sexual harassment victimization than males in six out of nine comparison across all the three dimensions/subscales. The non-significant findings involved physical harassment in Grade 8 and 9 and public display in Grade 7.

**Table 1:** Descriptive statistics, estimated correlations and gender differences

Variables	1.	2.	3.	4.	5.	6.	7.	8.	9.	10.	11.
1. Somatic G7^a^	**–**	**0.89**	0.07	**0.44**	**0.36**	0.14	0.15	0.01	**0.29**	**0.19**	−0.03
2. Negativity G7	**0.81**	**–**	**0.24**	**0.30**	**0.39**	**0.21**	0.20	**0.20**	**0.23**	**0.16**	−0.02
3. Anhedonia G7	**0.42**	**0.59**	**–**	**0.26**	**0.27**	**0.54**	0.07	0.01	**0.23**	**0.22**	0.13
4. Somatic G8	**0.73**	**0.51**	**0.39**	**–**	**0.81**	**0.15**	**0.50**	**0.35**	**0.20**	**0.17**	**0.25**
5. Negativity G8	**0.45**	**0.46**	**0.34**	**0.82**	**–**	**0.27**	**0.35**	**0.44**	**0.16**	**0.15**	**0.20**
6. Anhedonia G8	**0.26**	**0.32**	**0.54**	**0.38**	**0.49**	**–**	**0.16**	**0.24**	**0.50**	**0.21**	0.00
7. Somatic G9	**0.45**	**0.42**	**0.30**	**0.61**	**0.51**	**0.30**	**–**	**0.85**	0.09	0.00	**0.16**
8. Negativity G9	**0.41**	**0.56**	**0.43**	**0.45**	**0.48**	**0.34**	**0.81**	**–**	**0.27**	0.00	0.13
9. Anhedonia G9	**0.20**	**0.25**	**0.53**	**0.39**	**0.43**	**0.66**	**0.43**	**0.53**	**–**	0.03	−0.10
10. SH Physical G7	**0.53**	**0.46**	**0.26**	**0.44**	**0.25**	0.09	**0.23**	**0.22**	0.06	**–**	0.10
11. SH Physical G8	**0.47**	**0.39**	0.03	**0.48**	0.37	0.09	**0.25**	**0.27**	0.03	**0.66**	**–**
12. SH Physical G9	**0.36**	**0.22**	0.13	**0.37**	**0.32**	**0.13**	**0.40**	**0.30**	**0.13**	**0.63**	**0.61**
13. SH Name-call G7	**0.49**	**0.51**	**0.35**	**0.50**	**0.26**	**0.14**	**0.32**	**0.33**	**0.15**	**0.75**	**0.47**
14. SH Name-call G8	**0.28**	**0.25**	0.12	**0.43**	**0.34**	**0.14**	**0.35**	**0.34**	0.09	**0.43**	**0.67**
15. SH Name-call G9	**0.33**	**0.20**	0.11	**0.47**	**0.29**	**0.31**	**0.46**	**0.37**	0.24	0.14	**0.44**
16. SH Publ displ G7	**0.34**	**0.32**	0.10	**0.18**	0.10	**0.09**	**0.25**	**0.20**	−0.04	**0.72**	**0.37**
17. SH Publ displ G8	**0.31**	**0.31**	−0.10	**0.23**	**0.17**	0.06	0.05	0.13	0.03	**0.38**	**0.74**
18. SH Publ displ G9	**0.33**	0.08	−0.06	**0.19**	0.14	0.02	**0.27**	**0.23**	0.11	0.05	**0.22**
19. Migrant backg BL	0.06	0.03	0.06	0.08	0.04	−0.01	0.09	0.08	0.05	0.05	**0.19**
20. Disrupt family BL	−**0.27**	−**0.21**	−**0.15**	−**0.19**	−**0.11**	−**0.11**	−0.07	−0.07	−0.03	−**0.19**	−**0.09**
21. ≥1 par unempl BL	**0.14**	**0.18**	**0.21**	**0.16**	**0.15**	0.04	**0.14**	**0.17**	**0.16**	0.07	**0.11**
22. Rel affluence BL	**0.32**	**0.33**	**0.39**	**0.26**	**0.21**	**0.25**	**0.21**	**0.23**	**0.24**	**0.12**	**0.12**
Female (M, %)^b^	0.61	0.60	1.23	0.72	0.68	1.28	0.77	0.74	1.25	1.24	1.33
Female (SD)	0.35	0.23	0.50	0.39	0.21	0.44	0.47	0.18	0.35	0.12	0.14
Male (M, %)^b^	0.40	0.30	1.08	0.51	0.36	1.18	0.51	0.35	1.14	1.17	1.23
Male (SD)	0.45	0.13	0.34	0.40	0.05	0.24	0.44	0.09	0.14	0.04	0.07
Gender diff *p* level	<.001	<.001	<.001	0.147	<.001	0.644	<.001	<.001	0.128	0.029	0.068

Variables	12.	13.	14.	15.	16.	17.	18.	19.	20.	21.	22.
1. Somatic G7^a^	0.30	**0.32**	**0.19**	**0.34**	0.04	−0.04	**0.47**	**0.15**	−0.08	0.10	0.11
2. Negativity G7	0.15	**0.29**	**0.16**	0.21	0.06	−0.06	0.18	**0.16**	−**0.11**	0.09	**0.16**
3. Anhedonia G7	−0.19	**0.14**	**0.21**	−0.07	**0.26**	0.10	−0.07	0.06	−0.02	−0.05	**0.10**
4. Somatic G8	**0.24**	**0.25**	**0.33**	**0.24**	0.06	**0.21**	**0.31**	**0.14**	−**0.11**	0.08	**0.23**
5. Negativity G8	**0.18**	**0.21**	**0.25**	**0.23**	0.08	**0.17**	**0.19**	**0.20**	−**0.13**	**0.17**	**0.21**
6. Anhedonia G8	0.06	0.09	0.07	0.04	**0.20**	0.03	0.08	0.04	0.03	−0.01	**0.19**
7. Somatic G9	**0.30**	0.04	0.13	**0.29**	−**0.06**	0.06	**0.22**	**0.19**	0.02	0.07	**0.23**
8. Negativity G9	**0.33**	−0.05	**0.15**	**0.27**	−0.13	0.12	**0.25**	**0.18**	−0.01	**0.12**	**0.28**
9. Anhedonia G9	**0.19**	0.06	−0.06	0.10	−0.08	−**0.12**	**0.23**	**0.16**	−0.06	0.06	**0.19**
10. SH Physical G7	−0.05	**0.65**	**0.34**	0.12	**0.79**	0.03	−0.10	0.03	0.00	0.04	**0.13**
11. SH Physical G8	**0.19**	**0.14**	**0.60**	0.13	0.02	**0.88**	0.09	**0.14**	0.05	−0.05	0.00
12. SH Physical G9	**–**	0.06	**0.20**	**0.63**	−0.40	0.04	**0.91**	**0.15**	−0.08	0.09	**0.21**
13. SH Name-call G7	**0.43**	**–**	**0.49**	**0.33**	**0.51**	0.02	0.03	**0.12**	−0.06	**0.12**	**0.11**
14. SH Name-call G8	**0.55**	**0.57**	**–**	**0.40**	**0.23**	**0.49**	0.13	**0.15**	−0.03	0.01	0.06
15. SH Name-call G9	**0.65**	**0.28**	**0.65**	**–**	−0.08	0.11	**0.54**	**0.13**	−0.06	0.06	**0.14**
16. SH Publ displ G7	**0.39**	**0.52**	**0.20**	0.01	**–**	0.00	−0.36	−0.04	0.02	0.00	0.08
17. SH Publ displ G8	**0.33**	**0.26**	**0.55**	**0.20**	**0.47**	**–**	0.00	0.10	0.01	−0.03	0.01
18. SH Publ displ G9	**0.62**	0.24	**0.25**	**0.52**	−0.02	**0.11**	**–**	0.09	−0.07	**0.11**	**0.19**
19. Migrant backg BL	0.06	0.07	**0.11**	0.03	0.00	0.12	0.07	**–**	−0.05	**0.17**	**0.13**
20. Disrupt family BL	−0.05	−**0.21**	−**0.11**	−0.07	−0.07	−0.06	−0.01	−**0.08**	**–**	−**0.29**	−**0.14**
21. ≥1 par unempl BL	0.05	0.07	0.09	**0.15**	0.05	0.00	**0.11**	**0.19**	−**0.20**	**–**	**0.13**
22. Rel affluence BL	**0.11**	**0.21**	**0.11**	**0.12**	0.07	0.03	0.11	0.06	−**0.19**	**0.24**	**–**
Female (M, %)^b^	1.33	1.41	1.60	1.37	1.05	1.06	1.06	16.5	36.8	20.8	1.75
Female (SD)	0.15	0.06	0.20	0.22	0.04	0.06	0.06	–	–	–	0.89
Male (M, %)^b^	1.21	1.21	1.43	1.63	1.07	1.13	1.12	13.7	35.5	19.2	1.70
Male (SD)	0.07	0.23	0.22	0.08	0.02	0.03	0.03	–	–	–	0.96
Gender diff *p* level	0.104	<.001	<.001	<.001	.553	<.001	.020	.061	0.522	.335	.202

Estimated correlations for males on the bottom diagonal, females on the top diagonal

Significant correlations in bold (*p* ≤ .05)

^a^G7 = grade seven

^b^Descriptive statistic report the empirical means or frequency. To increase sensitivity gender differences were calculated using the estimated data which adjust for missing data

Females n = 1138, males n = 1192

The estimated correlations between dimensions of depressive symptoms, sexual harassment and covariates are also reported in Table 1. Significant correlations are shown in bold. The lower and upper diagonal shows the results for girls and boys respectively. Covariates were related to outcome variables in the expected manner. For example, living with both parents was for the most part inversely related to depressive symptoms and sexual harassment subscales, while having a migrant family background or unemployed parents were positively correlated with both outcomes although all these effects were small. In contrast, low personal relative affluence was moderately associated with depression and to a much lesser degree sexual harassment. In general, the patterns of correlation within each gender were similar, if not identical in magnitude.

### Subscale Directional Pathways

The initial overall model with all three dimensions of depressive symptoms and sexual harassment included showed satisfactory fit (CFI > 0.90), but did not show any overall difference between models despite significant cross-loadings. Accordingly, a series of models decomposing all three dimensions of depressive symptoms and sexual harassment was compared (Fig. 1). For each subscale combination, the Stability, SH → DS, DS → SH, and bidirectional pathways were compared in males and females separately rendering 72 smaller models (9 dimensions × 4 pathways × 2 genders). All of the compared models provided excellent fit to the data (CFI ≥ 0.95, full details available upon request). Table 2 summarizes the findings and indicates for each subscale combination which of our four pathways models had the most support, i.e. the best model fit. Public sexual harassment was unrelated to depression symptoms in both genders. Among boys, somatic, negative and anhedonia symptoms preceded later name-calling. The pattern among girls is mixed but suggests that physical sexual harassment are linked to future somatic symptoms in a reciprocal manner, and that name-calling precedes somatic symptoms as well as negative affect. Also, in girls, anhedonia preceded later name-calling.

**Table 2:** Comparison of dimensions of sexual harassment victimization and depressive symptoms; simple cross-lagged models by gender

Sexual harassment victimization	Depressive symptoms		
	Somatic	Negative	Anhedonia
*Female*			
Physical	B	O	O
Name-calling	SH	SH	D
Public display	O	O	O
*Male*			
Physical	O	O	O
Name-calling	D	D	D
Public display	O	O	O

O = Stability model; B = Bidirectional; SH = Sexual harassment > dep; D = Dep > SH

Mediation of the pathways between physical sexual harassment and name calling and the three depression subscales (somatic, negative and anhedonia) was tested using the “INDIRECT” option in Mplus. This is analogous to the Sobel mediation test used in conventional regression analysis and tests both direct and indirect pathways. The results mirror those shown in Table 2 where the cross-lagged models suggest the pathways between physical sexual harassment and somatic symptoms among females are bidirectional. In contrast, our tests for mediating pathways was non-significant. This discrepancy between these two findings suggests there is at least two distinct female subgroups with different vulnerabilities; one in which physical sexual harassment leads to future depression and a second showing the reverse pattern. For name-calling there were several significant mediation pathways between name-calling and future somatic, negative and anhedonia symptoms in girls. This matches the cross-lagged findings, suggesting a single group in regards to verbal harassment and subsequent depression. Among males, no significant mediation pathways were found between physical and anhedonia symptoms. However, in all other comparisons at least one significant mediating pathway was found between name-calling and physical sexual harassment to future depressive symptoms. This is broadly compatible with the cross-lagged models and suggests boys who experience depressive symptoms subsequently experience sexual harassment. The full results of all mediation models are available upon request.

### Combined Directional Pathways

Given the subscale findings, we re-specified the overall model by omitting public display sexual harassment subscale. Table 3 reports the fits for each of the four possible overall models: the basic stability pathway models; the SH → DS pathway model; the DS → SH pathway model; and the bidirectional model without the public display sexual harassment subscale.

**Table 3:** Fit indices of hypothesized cross-lagged models of sexual harassment and depressive symptoms over 1-year by gender

Models	Fit measures (Robust MLR estimator)					
	χ^2^	Df	CFI	RMSEA	∆χ^2^	*p* level^2^
*Adjusted female cross-lagged pathways* ^1^						
Stability (baseline)	2493.9	1827	0.94	0.018	–	–
Sexual harassment > depressive symptoms	2460.6	1815	0.94	0.017	33.3	<.001
Depressive symptoms > sexual harassment	2468.0	1815	0.94	0.017	25.9	<.001
Full cross-lagged	2440.3	1803	0.94	0.017	53.6	<.001
*Adjusted male cross-lagged pathways* ^1^						
Stability (baseline)	2554.8	1827	0.93	0.019	–	–
Sexual harassment > depressive symptoms	2539.8	1815	0.93	0.019	15.0	.855
Depressive symptoms > sexual harassment	2542.3	1815	0.93	0.019	12.5	.943
Full cross-lagged	2531.7	1803	0.93	0.019	23.5	.989

^1^Adjusted for: Personal relative affluence, parent(s) unemployed, migrant background, disrupted family

^2^Satorra–Bentler scaled χ^2^ difference test

All models show an acceptable fit. In girls, all other models were superior to the stability model with the reciprocal model providing the best fit in terms of the lowest Chi square (Table 3). In boys, all models also provided an acceptable fit, however, none of the models were statistically different from the stability model. Thus, the most parsimonious view is that both the targeting of students with elevated depressive symptoms and the consequence of sexual harassment explain a significant part of the relationship between depressive symptoms and sexual harassment victimization among girls. Among boys, only consequences of depressive symptoms explain the relationship between depressive symptoms and name-calling.

Table 4 shows the standardized pathway parameters estimates from the hypothesized models. These parameters provide an estimate of the relative effect size of the pathways and are interpretable as standardized correlation coefficients. Firstly, the stability pathways for sexual harassment victimization (*r* = 0.50–.64) and depressive symptoms (0.38–.61) were reasonably stable among girls. The corresponding pathways for boys were less stable (depressive symptoms, 0.38–.50; sexual harassment victimization, 0.10–.40). Notably, the continuity of physical sexual harassment was extremely low among boys, but verbal name calling harassment was enduring. Among girls, and consistent with the reciprocal model, several cross-lagged pathways between dimensions of sexual harassment and depressive symptoms were significant and in both directions (0.19–.39). Notably, in girls, the pathway between name-calling and somatic symptoms appears strong and consistent across grades. In boys, none of the cross-lagged pathways were significant. Formal multi-group comparisons confirmed that the models for girls and boys differed regarding the combined stability, covariation and cross-lagged pathways estimates, **∆**χ^2^ = 711.6, *p* < .001, which supports our decision to perform gender separate analyses.

**Table 4:** Cross-lagged standardized path coefficients, latent sexual harassment and depressive symptoms over 1-year by gender

Full model path loadings^a^	Female		Male	
	G7 > G8	G8 > G9	G7 > G8	G8 > G9
*Stability*				
SH physical	.62***	.64***	.10	.24**
SH name-calling	.58***	.50***	.40***	.36***
Somatic	.56***	.51***	.38***	.50***
Negative affect	.48***	.38***	.39***	.45***
Anhedonia	.52***	.61***	.50***	.47***
*Cross-lagged (significant)*				
SH name-calling > somatic	.24*	.25*	.09	−.11
SH name-calling > negative effect	.03	.38*	.09	−.02
Somatic > SH name-calling	.14	.39*	.10	−.11
Anhedonia > SH name-calling	−.05	.19**	.19	−.08

^a^Full cross-lagged model, adjusted for: Personal relative affluence, parent(s) unemployed, migrant background, disrupted family. Only significant pathways shown

* *p* < .05; ** *p* < .01; *** *p* < .001

## Discussion

The associations between adolescent sexual harassment victimization and poor mental health outcomes have been firmly established (Espelage and Holt 2007; Gruber and Fineran 2008; Chiodo et al. 2009; Goldstein et al. 2007; Bucchianeri et al. 2014; Skoog et al. 2015). Yet, information on the directional pathways between sexual harassment and depressive symptoms is scarce. Also, knowledge about how dimensions of sexual harassment and depressive symptoms relate to each other over time is lacking in the literature. We aimed to fill this gap by using cross-lag analysis to estimate the directional relationship between dimensions of sexual harassment and depressive symptoms in adolescents in a three wave sample from the northern part of Sweden. The findings show that public sexual harassment was unrelated to all dimensions of depressive symptoms irrespective of gender. All other subscales of sexual harassment victimization and depressive symptoms were related to each other in the manner discussed below.

### Stability Over Time

The findings support our first hypothesis that sexual harassment victimization is stable over time in girls; girls who were subjected to sexual harassment in grade seven continued to experience sexual harassment the following 2 years, while there was weaker evidence of repeated experience of sexual harassment among boys except for name-calling. Also, females reported significantly higher levels of sexual harassment victimization than males in six out of nine comparisons across all the three dimensions of sexual harassment. Furthermore, we found support for stability over time in depressive symptoms in both genders, which is contrary to previous research (Cole et al. 2009; Abela and Hankin 2011) who found that depressive symptoms in boys are not consistent over time. Nevertheless, females reported significantly higher levels of depressive symptoms in males in six out of the nine subscale comparisons.

### Sexual Harassment Preceding Depressive Symptoms

Our second hypothesis (that dimensions of sexual harassment would precede dimensions of depressive symptoms if sexual harassment is a means of establishing asymmetric power relations) was partly supported in girls—sexual name-calling was associated with later somatic symptoms and negative affect. Sexual harassment was not associated to later depressive symptoms in boys. According to previous research, most perpetrators are male (Lichty and Campbell 2012; McMaster et al. 2002; Hill and Kearl 2011; Hand and Sanchez 2000) and within the poststructuralist feminist framework suggested in this study, name-calling may be the means by which (male) perpetrators try to make (female) peers vulnerable, and hence establish asymmetric power relations.

### Depressive Symptoms Preceding Sexual Harassment

There was limited support of this directional pathway in girls as only anhedonic depressive symptoms preceded later name-calling. In boys, anhedonia also preceded sexual name-calling suggesting that both girls and boys suffering from anhedonia are vulnerable to, or easy targets of, sexual name-calling. Hence, our findings suggest that an anhedonic predisposition may be a risk factor for sexual harassment victimization in both genders. Gilbert et al. (2002) identified that anhedonia is associated with social withdrawal, shaming experiences, defeat and entrapment processes. They did not, however, address the directions of these associations (Gilbert et al. 2002). Furthermore, in boys, somatic symptoms and negative affect preceded sexual name-calling which further suggests that vulnerable boys are targets of name-calling victimization. This supports the hypothesis that the targeting of vulnerable boys, mainly by other boys, can be interpreted as a way of displaying male dominance by emphasizing hegemonic masculine stereotypes of strength and toughness (Lichty and Campbell 2012; McMaster et al. 2002; Hill and Kearl 2011; Hand and Sanchez 2000; Petersen and Hyde 2009; Conroy 2013; Kimmel 2008). Boys may be particularly vulnerable to sexual harassment victimization (in the form of name-calling) if they display depressive symptoms and signs of vulnerability since this is not associated with hegemonic masculinity. In a cross-lag study by Sweeting et al. (2006), they showed that the pathways between bullying and depression in 15-year-old boys and which mirrors the findings in our study, i.e. the higher level of depressive symptoms, the higher the risk of bullying victimization. In general, bullying is not usually considered a type of gendered violence, however, the targeting of vulnerable boys may, according to a feminist theoretical approach, be interpreted as a way of displaying hegemonic male dominance within male peer groups (Conroy 2013; Kimmel 2008). A similar process may operate in both bullying and sexual harassment victimization, but the effects of sexual harassment on boys are weak compared to that of bullying, possibly because of the transient nature of sexual harassment experienced by boys.

### Bidirectional Pathways

The relationship between dimensions of sexual harassment victimization and depressive symptoms was hypothesized to be bidirectional if sexual harassment perpetration is used as a means to both establish and reinforce asymmetric power relations. The analyses showed limited support for this hypothesis, as only physical sexual harassment had a bidirectional relationship with somatic symptoms in girls. While somatic symptoms may be interpreted as a form of embodiment of physical harassment experiences, the reversed direction, i.e., why girls with somatic symptoms are at risk for physical sexual harassment is more difficult to interpret and calls for further investigation. This finding is in line with Marshall et al. (2013) who suggested that the directional pathways of sexual harassment victimization and self-harm are bidirectional among adolescent girls. Self-harming behaviors have also been linked to embodiment processes or body-based experiences as shown by Horne and Csipke (2009).

### A Feminist Theoretical Approach

A dominant discourse in understanding adolescent sexual harassment is that sexual harassment perpetration may be a nascent attempt by adolescents to express sexual or romantic interest (AAUW 2001), which they fail to do in a socially appropriate manner (McMaster et al. 2002; Petersen and Hyde 2009). Alternatively, it is seen as the consequence of moving from single-gender to cross-gender peer networks, which some adolescents have not been sufficiently prepared for (McMaster et al. 2002). McMaster et al. (2002) also discuss that, in elementary and middle school, sexual harassment perpetration may be a time-limited misbehavior or deviance and an individual’s response to the stress of coping with the changes that occur in adolescence. The same authors also propose that, for a different group of adolescents, sexual harassment perpetration may be a part of a more general harassment behavior with origins earlier in development (McMaster et al. 2002). Employing the perspective that adolescent sexual harassment perpetration is about sexual or romantic interest (Petersen and Hyde 2009; McMaster et al. 2002) is, however, of little help when interpreting the findings of the present study; it does, for example, not explain why vulnerable individuals are targeted. Petersen and Hyde 2009 also reported that girls and boys with high perceived power were more likely to be sexually harassed by peers with less perceived power as they would use such behavior as means to gain attention from more powerful cross-gender peers. For example, girls in grade nine who were perpetrators were more likely to harass powerful male peers than those less powerful (Petersen and Hyde 2009). Given the association between depressive symptoms and sexual harassment in our study, this would imply that youth, and especially boys, who have elevated depressive symptoms also perceive themselves as powerful. This seems implausible as adolescent depressive symptoms are highly associated with both low global (Sowislo and Orth 2013; Zetterström Dahlqvist et al. 2012) and low contingent (including social acceptance and approval) self-esteem (Bos et al. 2010). In fact, subjective adolescent peer social status, which includes perceived power as a subcomponent, is associated with *better* mental health (Sweeting and Hunt 2014). In the present study there was no information available on perceived power, but further investigations might illuminate whether this could explain why there are few mental health consequences for boys who are victimized. Furthermore, as Felix and McMahon (2007a) have shown, being victimized by a girl did not have any impact on internalizing or externalizing behaviors regardless of the victim’s gender. It has also been reported that when girls are perpetrators, it is the result of victimization, i.e., acts of retaliation, while for boys the opposite is true, i.e., boys who perpetrate sexual harassment subsequently become victims (Fineran and Bolen 2006). Consequently, we argue that a feministic theoretical approach is a fruitful approach to understanding and clarifying the direction of these associations, because focus is directed towards understanding sexual harassment as enactment of power and gender relations. Again, it is important to acknowledge that, even if both girls and boys can be perpetrators, sexual harassment perpetrators are predominantly males (Lichty and Campbell 2012; McMaster et al. 2002; Hill and Kearl 2011; Hand and Sanchez 2000). Cognizant of the existing research, our results are compatible with a model of sexual harassment which proposes that the majority of perpetrators use sexual harassment as a ways of asserting dominance towards vulnerable peers, males and females alike, and, establishing asymmetric power relations between the genders. This is similar to other forms of harassment proposing that, i.e., bullies, target peers who transgress gender norms (Young and Sweeting 2004).

### Methodological Considerations

In the present study, the models for girls provided far greater explanatory power than those for boys. This is mainly due to the poor stability of sexual harassment among boys. Accordingly, controlling for exogenous confounders makes our conclusions more plausible. In addition, the initial overall model with all three dimensions of sexual harassment and depressive symptoms showed acceptable fit, but did not show any overall difference between models irrespective of gender. When we deconstructed our models into subscales of sexual harassment victimization and depressive symptoms, another picture emerged with directional pathways significantly different from the stability model. This is a strength in the present study because this gives important information about the relationship between the studied variables. A related issue is that we do not know if the sexual harassment victimization in these age groups is an “occasional” or “persistent” exposure, i.e., part of everyday life since we study experiences during the last 6 months. Previous research has, however, shown that it is likely to be more than just occasional (deLara 2008; Witkowska and Menckel 2005; Hlavka 2014).

Cross-lagged analysis has a number of assumptions, such as selecting an appropriate time lag between measures (Little et al. 2007), but if these are accepted, this method allows us to draw conclusions about the consequences of long-term sexual harassment on girls’ mental health. That said, selecting a shorter time lag would likely increase the stability between measure points and the strength of cross-lagged correlations. In addition, having had access to data on both perpetration and victimization of sexual harassment may have elucidated the relationships between sexual harassment and depressive symptoms further, as would the study of vulnerable populations such as sexual minority students. There is considerable evidence that harassment, sexual or otherwise, impacts the mental health of vulnerable boys who transgress gender norms (Young and Sweeting 2004), which may be masked in this large community study.

Another methodological consideration is missing data and the non-normal distributions of both depressive symptoms and sexual harassment. By employing RFMLE, potential biases are addressed in a statistically principled manner. A related issue is that girls lost to attrition from Wave 2 to Wave 3 had significantly higher levels of sexual harassment victimization. However, the accelerated panel design at least partially addresses this issue. While we adjusted for social background we may have omitted potentially important confounders or sources of bias such as school or classroom effects. Accelerated cohort designs can also be analyzed using multiple group methods, which under certain conditions provide less biased estimates (Little et al. 2007). Due to the design of the study, this was not possible, but a number of sensitivity analyses were carried out comparing estimates under different assumptions: unadjusted vs adjusted for confounders; baseline vs time-varying confounders; using different estimation methods; complete vs missing data; and adjusting for potential cohort effects. The reported results were substantively no different regarding interpretation and provided the most conservative estimates.

An inherent limitation in a cross-lagged design is that the tested models evaluate consistency of associations rather than individual levels of the measured constructs. This limits the insights that can be gleaned about the actual importance of each variable. This distinction is crucial to understanding the experience of boys in whom we could not show a directional path from any of the dimensions of sexual harassment victimization to any of the dimensions of depressive symptoms. A growth trajectory model approach would be feasible to understand this better (Preacher et al. 2008).

### Implications for Future Studies and Practice

Given the dearth of studies investigating the directional pathways between dimensions of sexual harassment and depressive symptoms, replication is warranted. Furthermore, future studies should also investigate whether sexual harassment victimization prolongs episodes of depressive symptoms since in girls as well as boys, this could be an important issue as different dimensions of depressive symptoms precede sexual harassment victimization. Recent research has concluded that interventions which shorten the duration of depressive episodes in adolescence could prevent morbidity later in life (Patton et al. 2014). Interventions against sexual harassment in general, and name-calling in particular, may in light of the results of the current study have the potential to shorten episodes of depressive symptoms in boys. In girls, interventions against sexual harassment in general and name-calling in particular may both prevent new episodes of depressive symptoms as well as shorten current episodes. Our findings could, therefore, potentially inform health promotion practice mainly in schools but also elsewhere. Those who take a developmental approach have recommended social and emotional interventions such as Social and Emotional Learning (SEL) (Collaborative for Academic Social and Emotional Learning 2015) to address the issue of sexual harassment in schools by promoting empathy and interpersonal competence (Skoog et al. 2015). While promoting empathy and interpersonal competence may be beneficent in this age group, we also recommend interventions that target sexual harassment as an assertion of male dominance and as a tool for policing (hetero)sexuality as suggested by, e.g., Conroy (2013) and Robinson (2005). Addressing sexual harassment with focus on name-calling in both genders, and also physical sexual harassment in girls, would protect vulnerable students from additional harm as well as protect youth from developing depressive symptoms in the first place. Also, it would help undermine socially constructed and privileged notions of heterosexual masculinity and would most likely be beneficial to girls and boys alike.

## Conclusions

The findings of this longitudinal study provide new insights regarding the directional pathways between dimensions of sexual harassment and depressive symptoms in adolescent girls and boys. Sexual harassment victimization was stable over time in girls while there was weak evidence of stability of sexual harassment in boys. In contrast to previous research (Cole et al. 2009; Abela and Hankin 2011), we found evidence for stability over time in depressive symptoms for both genders. Our second hypothesis, that dimensions of sexual harassment precede dimensions of depressive symptoms if sexual harassment is associated with establishing asymmetric power relations between genders, was partly supported in girls—sexual name-calling was associated with later somatic and negative affect symptoms in girls. In girls, there was limited support for our third hypothesis (a directional pathway from depressive symptoms to sexual harassment) as anhedonia depressive symptoms to name-calling only had support in girls. In boys, anhedonia also preceded sexual name-calling, suggesting that both girls and boys suffering from anhedonia are vulnerable to name-calling. Also, in boys, somatic symptoms and negative affect preceded sexual name-calling, suggesting that vulnerable boys may be at risk of name-calling victimization. A bidirectional relationship between dimensions of sexual harassment victimization and depressive symptoms had only limited support in our data as only physical sexual harassment had a bidirectional relationship to somatic symptoms in girls.

Our study contributes to the field of research on the important developmental period of adolescence in mainly two ways: Firstly, the feminist framework that proposes that sexual harassment is a tool to establish asymmetric power relations and to assert hegemonic (usually heterosexual) masculinity (Kimmel 2008) may help shed some light on who is predisposed to what type of sexual harassment victimization and why sexual harassment victimization has different consequences for girls and boys. Girls are both vulnerable as a result of victimization, which is a way for male peers to establish asymmetric power relations, as suggested by, for example, Robinson (2005) and Jones and Mahony (1989), and being victimized *because* they are vulnerable, which is a way to reinforce asymmetric power relations as suggested by for example Robinson (2005) and Conroy (2013). Boys are only victimized when already vulnerable and as a boy, being vulnerable is not concordant with hegemonic heterosexual masculinity (Conroy 2013; Robinson 2005). The results also showed that there were no consequences of sexual harassment victimization in boys without a vulnerable predisposition. Secondly, information on what specific dimensions of sexual harassment and depressive symptoms that are involved in the directional relationships studied here may inform future interventions to prevent depressive symptoms as well as prevent sexual harassment perpetration.
